# Nitrogen-doped mesoporous activated carbon from *Lentinus edodes* residue: an optimized adsorbent for pharmaceuticals in aqueous solutions

**DOI:** 10.3389/fchem.2024.1419287

**Published:** 2024-06-20

**Authors:** Bei Chu, Yichen Lou, Yixin Tan, Jiawei Lin, Xingcheng Liu

**Affiliations:** Ningbo Key Laboratory of Agricultural Germplasm Resources Mining and Environmental Regulation, College of Science and Technology, Ningbo University, Cixi, China

**Keywords:** *Lentinus edodes* residue, N-doped activated carbon, mesoporous, adsorption, acetaminophen, carbamazepine, metronidazole

## Abstract

In this study, phosphoric acid activation was employed to synthesize nitrogen-doped mesoporous activated carbon (designated as MR1) from *Lentinus edodes* (shiitake mushroom) residue, while aiming to efficiently remove acetaminophen (APAP), carbamazepine (CBZ), and metronidazole (MNZ) from aqueous solutions. We characterized the physicochemical properties of the produced adsorbents using scanning electron microscopy (SEM), nitrogen adsorption isotherms, and X-ray photoelectron spectroscopy (XPS). MR1, MR2, and MR3 were prepared using phosphoric acid impregnation ratios of 1, 2, and 3 mL/g, respectively. Notably, MR1 exhibited a significant mesoporous structure with a volume of 0.825 cm^3^/g and a quaternary nitrogen content of 2.6%. This endowed MR1 with a high adsorption capacity for APAP, CBZ, and MNZ, positioning it as a promising candidate for water purification applications. The adsorption behavior of the contaminants followed the Freundlich isotherm model, suggesting a multilayer adsorption process. Notably, MR1 showed excellent durability and recyclability, maintaining 95% of its initial adsorption efficiency after five regeneration cycles and indicating its potential for sustainable use in water treatment processes.

## 1 Introduction

Industrial effluent and urban sewage discharge are increasing at a rapidly due to the acceleration of industrial development and urbanization. Water pollution is a major obstacle to the development of industrial green transformation and the efficient use of water resources. The usage of pharmaceutical and personal care products (PPCPs), which predominantly comprise antibiotics and antipyretics, is expanding in line with their prevalence in daily life (Luo et al., 2023). Concurrently, inadequate infrastructure of treatment facilities contributes to the increasing complexity of water quality associated with the discharge of urban wastewater (Kujawska et al., 2022). Thus, it is imperative to eliminate PPCPs from contaminated wastewater and drinking water to protect human health and the environment (Li et al., 2022).

Various methods are used for the removal of PPCPs including advanced oxidation process (AOPs) (Kumar et al., 2023; Quan et al., 2023), biodegradation (Liu et al., 2022; Du et al., 2023), membrane separation (Lin et al., 2023; Li et al., 2024), and adsorption (Jin et al., 2018; Yin et al., 2020). Among them, advanced oxidation methods may result in incomplete degradation of pollutants and the production of more toxic compounds, while membrane separation methods have higher costs (Kodali et al., 2023a). Adsorption is an effective and environmentally friendly approach for eliminating organic contaminants from wastewater (Wu et al., 2020; Kodali et al., 2023b). This method employs physical and chemical adsorption interactions between the adsorbent and organic pollutants, causing the pollutants to adhere to the internal pores or surfaces of the adsorbent, thereby eliminating organic pollutants from water (Kodali et al., 2021; Wang et al., 2022). In addition to a high removal efficiency, this method is also characterized by its ease of operation, low energy consumption, and absence of toxic by-products (Yao et al., 2022). Activated carbon has high specific surface area and porous structure and is presently the most widely used adsorbent (Zhu et al., 2022). It effectively removes most typical dissolved organic compounds. In particular, the removal of PPCPs from wastewater relies primarily on intermolecular physicochemical action (Asif et al., 2021). Adsorbents with wide applicability, including zeolites, molecular sieves, and activated carbon, typically possess a specific surface area (Zhu et al., 2016).

Porous activated carbon, characterized by its hierarchical cavities and a rich array of surface functional groups, primarily consists of carbon (C), oxygen (O), nitrogen (N), and sulfur (S) elements. This material is available in various forms, including powdered, block, granular, and honeycomb configurations. Pore sizes are categorized into mesopores (2–50 nm), micropores (<2 nm), and macropores (>50 nm) according to the classifications set forth by the International Union of Pure and Applied Chemistry (IUPAC). The adsorptive capability of porous activated carbon is greatly influenced by its pore architecture and the spatial arrangement of these pores (Suresh Kumar et al., 2017). A high specific surface area, coupled with a dense and evenly distributed pore network, enhances the efficiency of pollutant uptake by providing numerous active sites for adsorption (Lei et al., 2006). Micropores contribute to this process by providing an array of potential adsorption sites, while mesopores serve as conduits that facilitate the movement of adsorbates, thereby enhancing the rate of adsorption (Wang et al., 2023). Mesopores also directly sequester larger molecular entities, such as organic pollutants (Shao et al., 2022). Despite the widespread use of mesoporous activated carbon for adsorption processes, research on its synthesis remains sparse (Wu et al., 2023; Shi et al., 2024). Consequently, exploring alternative methods for producing mesoporous activated carbon is crucial.

Increasing interest in nitrogen doping technology stems from its ability to modify the surface of porous carbon, thereby amplifying the surface chemical properties of the material (Xiong et al., 2023). Nitrogen atoms, distinguished by their high electronegativity and structural resemblance to carbon atoms, imbue activated carbon with distinct electronic properties and increase the number of surface functional groups (Qu et al., 2022). This enhances the performance of the carbon material (Gao et al., 2022). Notably, nitrogen-doped activated carbon has been shown to efficiently remove organic pollutants (Kasera et al., 2022). The process of *in situ* nitrogen doping involves the direct carbonization and activation of nitrogen-containing substances or nitrogen-rich biomass (Zhang X. et al., 2023). This approach is operationally straightforward, eliminates the need for supplemental nitrogen sources, and is considered a more environmentally friendly method of nitrogen doping (Chen et al., 2022). Given its rich content of cellulose, hemicellulose, lignin, and protein, the residue of *Lentinus edodes*, the second most cultivated edible fungus in the world, is an ideal substrate for producing nitrogen-doped activated carbon.

The present study presents the fabrication of nitrogen-doped mesoporous activated carbon utilizing the residue of *Lentinus edodes*, using phosphoric acid as the activating agent. MR1, MR2, and MR3 were prepared using phosphoric acid impregnation ratios of 1, 2, and 3 mL/g, respectively. The synthesized material MR1 exhibited a high proficiency in the adsorptive removal of acetaminophen (APAP), carbamazepine (CBZ), and metronidazole (MNZ). The study aimed to elucidate the adsorption isotherms and kinetic behaviors of these organic contaminants on the nitrogen-enriched mesoporous carbon structure. Furthermore, this study compared the adsorption capabilities of nitrogen-doped mesoporous activated carbon (MR1) with those of other adsorbents. The chemical functional groups and surface physical properties of the nitrogen-doped mesoporous activated carbon were characterized to elucidate its adsorption mechanism. The primary objective of this work was to measure the adsorption interactions between organic pollutants and the nitrogen-doped mesoporous activated carbon derived from *Lentinus edodes* residue. The findings of this study establish a theoretical foundation for the application of biomass-derived activated carbons in environmental remediation.

## 2 Materials and methods

### 2.1 Preparation of nitrogen-doped mesoporous activated carbon

The *Lentinus edodes* residue was obtained from a farm in Cixi, China. The *Lentinus edodes* residue was washed with distilled water and dried in an oven at 110°C. After drying, it was immersed in a 90°C NaOH solution (3 M) and kept at temperature for 3 h. Then, it was washed with HCl (0.1 M) and hot distilled water, and dried again in an oven overnight at 110°C. The alkali-treated *Lentinus edodes* residue (almost 1 g) was immersed in an oven at a ratio of 1 mL g^−1^ for 8 h using a H_3_PO_4_ solution (1 mL). The temperature inside the oven was set to 70°C to ensure sufficient H_3_PO_4_ penetration. Then, the mixture was dried overnight in an oven at 110°C. The resulting mixture was transferred to a porcelain boat and placed in a tube furnace heated to 500°C in a N_2_ gas atmosphere for 1 h with a N_2_ gas flow rate of 100 mL min^−1^. After carbonization, the porcelain boat was cooled to ambient temperature in a N_2_ gas atmosphere. After stirring the mixture in a 1 M HCl solution for 1 h, the prepared activated carbon was rinsed in a Soxhlet extractor for 2 h and dried overnight at 110°C. The final product was named MR1. The impregnation ratio of phosphoric acid was modified using a similar method, resulting in modified values of 2 and 3 mL g^−1^ for MR2 and MR3, respectively. For comparison, the shiitake mushroom residue was directly placed in a tube furnace and heated at 500°C for 1 h, which was referred to as MR.

### 2.2 Activated carbon characterization

The synthesized activated carbons were characterized via their mesoporous structure, surface area, and microporosity via nitrogen adsorption/desorption isotherms at −196°C, using a Micromeritics ASAP 2020 surface area analyzer (Micromeritics, Norcross, GA, United States). The morphological features were examined using a ZEISS Sigma 300 SEM (ZEISS Sigma 300, Oberkochen, Germany). Elemental analysis for carbon, hydrogen, nitrogen, and oxygen content was conducted using an Elementar UNICUBE analyzer (UNICUBE, Elementar, Langenselbold, Germany). Surface chemical compositions were probed by X-ray photoelectron spectroscopy (XPS) using a Thermo Scientific K-Alpha instrument (Thermo Scientific K-Alpha, Waltham, MA, United States). Post-experiment, the concentrations of carbamazepine, acetaminophen, and metronidazole in the solutions were quantified using an Agilent 1260 InfinityII HPLC (HPLC, Agilent 1260 Infinity II, Santa Clara, CA, United States). Furthermore, the surface functional groups of the adsorbents were evaluated using Fourier-transform infrared spectroscopy (FTIR) on a Thermo Scientific Nicolet iS20 spectrophotometer (Thermo Scientific Nicolet, United States). The zeta potential of samples was measured by zeta potential analyzer (Malvern Zetasizer Nano ZS90, United Kingdom).

### 2.3 Batch adsorption test

A stock solution containing 100 mg/L of APAP, CBZ, and MNZ was prepared by dissolving 100 mg of each pharmaceutical in 1,000 mL of deionized water. Adsorption experiments were conducted at 25°C using a batch equilibrium method in 50 mL Erlenmeyer flasks. For these tests, 0.02 g of the adsorbent was introduced to 20 mL of the solution with varying concentrations of the target compounds. The adsorption capacities for MNZ, APAP, and CBZ were quantified by the reduction in their concentrations in the solution, as defined by the Eq. 1:

$$Q_{e}=\left(C_{0}-C_{e}\right)\times V/m$$ (1)

where Q_e_ (mg g^−1^) is the equilibrium adsorption capacity for the pharmaceuticals on the activated carbons; m (g) is the mass of the adsorbent; V (L) is the volume of the solution; C_0_ (mg L^−1^) is the initial concentration of the pharmaceuticals; and C_e_ (mg L^−1^) is their equilibrium concentration post-adsorption.

Experimental procedures were conducted to ascertain the upper limit of adsorption for MNZ, APAP, and CBZ using activated carbon, a series of experiments were performed with each carbon at a concentration of 1 g L^−1^. We introduced the activated carbon into a 30 mL pharmaceutical mixture, ensuring a neutral pH balance of 7. Subsequently, we evaluated the adsorption potential of various carbon samples (MR, MR1, MR2, and MR3).

To investigate how pH variations influence the adsorption process for APAP, CBZ, and MNZ, we modified the pH of a 100 mg L^−1^ pharmaceutical solution to a spectrum of pH values from 2 to 9. This adjustment was achieved through the application of either 0.01 mol L^−1^ HCl or NaOH. pH measurements were accurately recorded using an INESA PHS-3C pH meter. Post a 24-h shaking period, we measured the final concentrations of the pharmaceuticals to establish equilibrium states.

We further explored the adsorption kinetics of APAP, CBZ, and MNZ on activated carbon by agitating 0.1 g of carbon with 100 mL of a 100 mg L^−1^ pharmaceutical solution. Sampling at intervals (5, 10, 20, 30, 40, 60, 120, 240, and 360 min) allowed for the concentration analysis over time. To decode the kinetic data, we applied both pseudo-first-order and pseudo-second-order models, with Eqs 2, 3 represented for clarity.

$$\ln\left(Q_{e}-Q_{t}\right)=\ln Q_{e}-k_{1}t,$$ (2)

$$\frac{t}{Q_{t}}=\frac{1}{k_{2}Q_{e}^{2}}+\frac{t}{Q_{e}},$$ (3)

where Q_e_ denotes the equilibrium adsorption capacity for APAP, CBZ, and MNZ (mg g^−1^) and Qt represents the quantity of these compounds adsorbed (mg g^−1^) at a given time t (min). The rate constants for the pseudo-second-order and pseudo-first-order kinetics are represented by k_2_ (g mg^−1^ min^−1^) and k_1_ (L min^−1^), respectively.

We investigated the surface properties of the activated carbon and its interaction dynamics with APAP, CBZ, and MNZ molecules by applying adsorption isotherm models to the collected data. The adsorption behavior of these drugs was determined across varying initial concentrations, spanning from 10 to 100 mg L^−1^. The Langmuir model, which posits that adsorption is a single-layered process on a consistent adsorbent surface, was one of the models used to interpret the data. Both the Langmuir and Freundlich isotherm models were utilized to elucidate the adsorption phenomena. The Langmuir model is adept at detailing both physisorption and chemisorption, particularly emphasizing the formation of a monolayer adsorption on a homogenous surface. The Langmuir isotherm equation’s linearity is depicted by Eq. 4.

$$\frac{C_{e}}{Q_{e}}=\frac{1}{X_{m}K_{L}}+\frac{1}{X_{m}}C_{e},$$ (4)

where Q_e_ (mg g^−1^) denotes the adsorbed amount of APAP, CBZ, and MNZ at equilibrium; X_m_ (mg g^−1^) is the monolayer maximum adsorption capacity; C_e_ (mg L^−1^) is the equilibrium concentration of APAP, CBZ, and MNZ in the solution; and K_L_ (L mg^−1^) is the adsorption energy related Langmuir constant.

An alternate approach to describe the adsorption interactions on non-uniform surfaces is provided by the Freundlich adsorption model. This model assumes that the adsorbent’s surface is energetically heterogeneous, with a varied range of binding site energies and affinities. The adsorption process, according to this model, involves a spectrum of different energy sites on the adsorbent. The empirical equation of the Freundlich model, which is often presented in a linear form for practical applications, is delineated as Eq. 5:

$$\ln Q_{e}=\ln K_{F}+\frac{1}{n}\ln C_{e},$$ (5)

where the Freundlich isotherm constant, K_F_, indicates the adsorption capacity, and 1/n, which serves as an indicator of adsorption intensity or surface heterogeneity, are integral. A larger n value signifies a more favorable adsorption process.

To evaluate the reusability of the activated carbon, we introduced 1,000 mg of the material into a 1,000 mL Erlenmeyer flask filled with an aqueous mixture containing APAP, CBZ, and MNZ, each at an initial concentration of 100 mg L^−1^. We adjusted the solution’s pH to 7. The mixture was then stirred at a speed of 100 revolutions per minute and maintained at a steady temperature of 25°C until it reached adsorption equilibrium after 24 h. Following this, we passed the mixture through a filter with a pore size of 0.45 μm. During the desorption stage, the filtered carbon was washed with distilled water and subsequently immersed in 1,000 mL of a methanol solution at a concentration of 10% for a duration of 12 h. We repeated this cycle of adsorption and desorption five times to comprehensively determine the material’s capacity for regeneration.

## 3 Results and discussion

### 3.1 Material characterization

**FIGURE 1:**
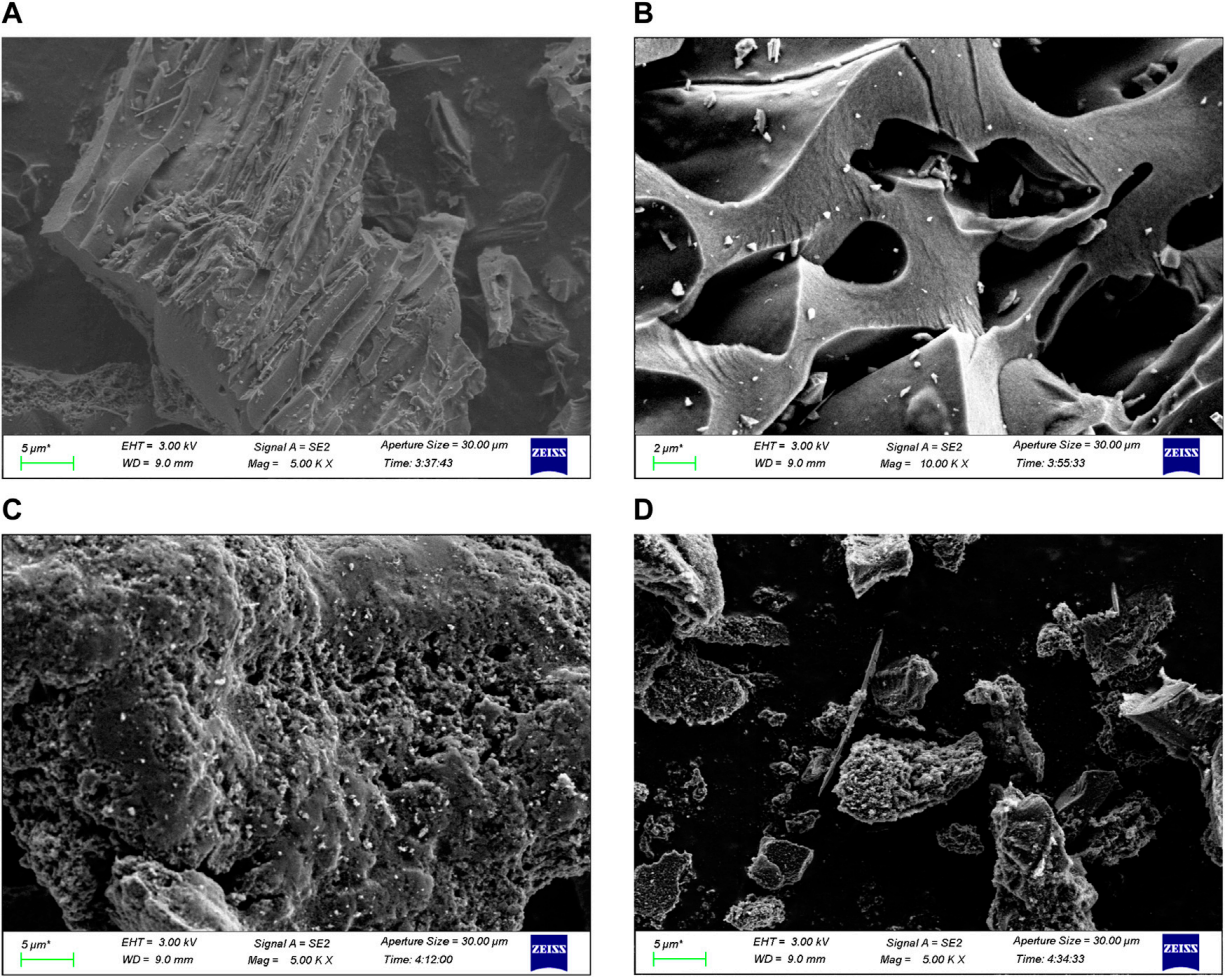
SEM images of **(A)** MR, **(B)** MR1, **(C)** MR2, and **(D)** MR3.

SEM images revealed that the shape of MR was discoid, with relatively flat sides and nearly no surface pores (Figure 1). MR1 had a fibrous surface with high porosity and a developed pore structure. The pore size was uneven, the density of distribution varied, and the surface structure was loose and irregular. The surface of MR2 exhibited a rough texture characterized by the presence of numerous small holes. SEM images of MR1 before and after adsorption of APAP, CBZ, and MZN were shown in Supplementary Figure S4. It is evident that the surface of MR1 remains very smooth before and after adsorption and maintains a porous structure, thereby indicating that none of these three pollutants can damage the structure of MR1. Adsorption capacity, chemical analysis, surface area, and pore volumes of the activated carbon samples were determined and evaluated, and the results are reported in Table 1. The initial specific surface area of MR samples was 43 m^2^ g^−1^, while the mesoporous volume and specific surface area of activated carbon were significantly improved after NaOH pretreatment and phosphoric acid activation of *Lentinus edodes* residue (Table 1). The specific surface area of MR1 was the highest, reaching 1,645.6 cm^2^ g^−1^, with the mesoporous volume of around 0.825 cm^3^ g^−1^. As the concentration of phosphoric acid increased during activation, there was a progressive decrease in the specific surface areas of MR2 and MR3, which reached 1,342.5 and 429.1 m^2^ g^−1^, respectively. Excess phosphoric acid led to pore combination and collapse, which ultimately resulted in a reduction in specific surface area (Cheng et al., 2023). The average diameter (D_avg_) of MR1 was 6.8 nm, demonstrating its mesoporous nature. The average pore sizes (D_avg_) of activated carbon MR1, MR2, and MR3 were 2.5, 5.0, and 7.5 nm, respectively, indicating mesoporous properties. MR1 had an average pore size of about 2 nm, and an N_2_ adsorption-desorption isotherm like the type I isotherm. In contrast the isotherms for MR2 and MR3 were like the type IV isotherm due to their larger average pore sizes. The tensile strength effect (TSE) phenomenon usually occurs in the desorption process, resulting in disparate N adsorption and desorption results (Yuan et al., 2021). The TSE of MR1 occurred in the p/p0 range of 0.35–0.7 (Supplementary Figure S1). The nitrogen content in MR1 and MR2 were found to be 2.6%, while MR3 exhibited a nitrogen content of 2.2%, surpassing that of the untreated MR, as shown in Table 1. This suggests that the NaOH pretreatment followed by phosphoric acid activation is an effective method for eliminating impurities from *Lentinus edodes* residue while efficiently preserving the nitrogen component.

**TABLE 1:** Textural and surface properties and elemental composition of each prepared activated carbon sample.

Sample	C	H	N	O	S_BET_	V_total_	V_micro_	V_meso_
	(%)	(%)	(%)	(%)	(m^2^ g^−1^)	(cm^3^ g^−1^)	(cm^3^ g^−1^)	(cm^3^ g^−1^)
MR	85.8	2.5	0.2	10.7	43.9	0.0411	0.011	0.029
MR1	75.0	2.6	2.6	17.5	1,645.6	0.926	0.101	0.825
MR2	67.0	2.5	2.6	12.7	1,342.5	1.77	0.003	1.769
MR3	23.0	0.7	2.2	9.5	429.1	0.626	0.027	0.598

**FIGURE 2:**
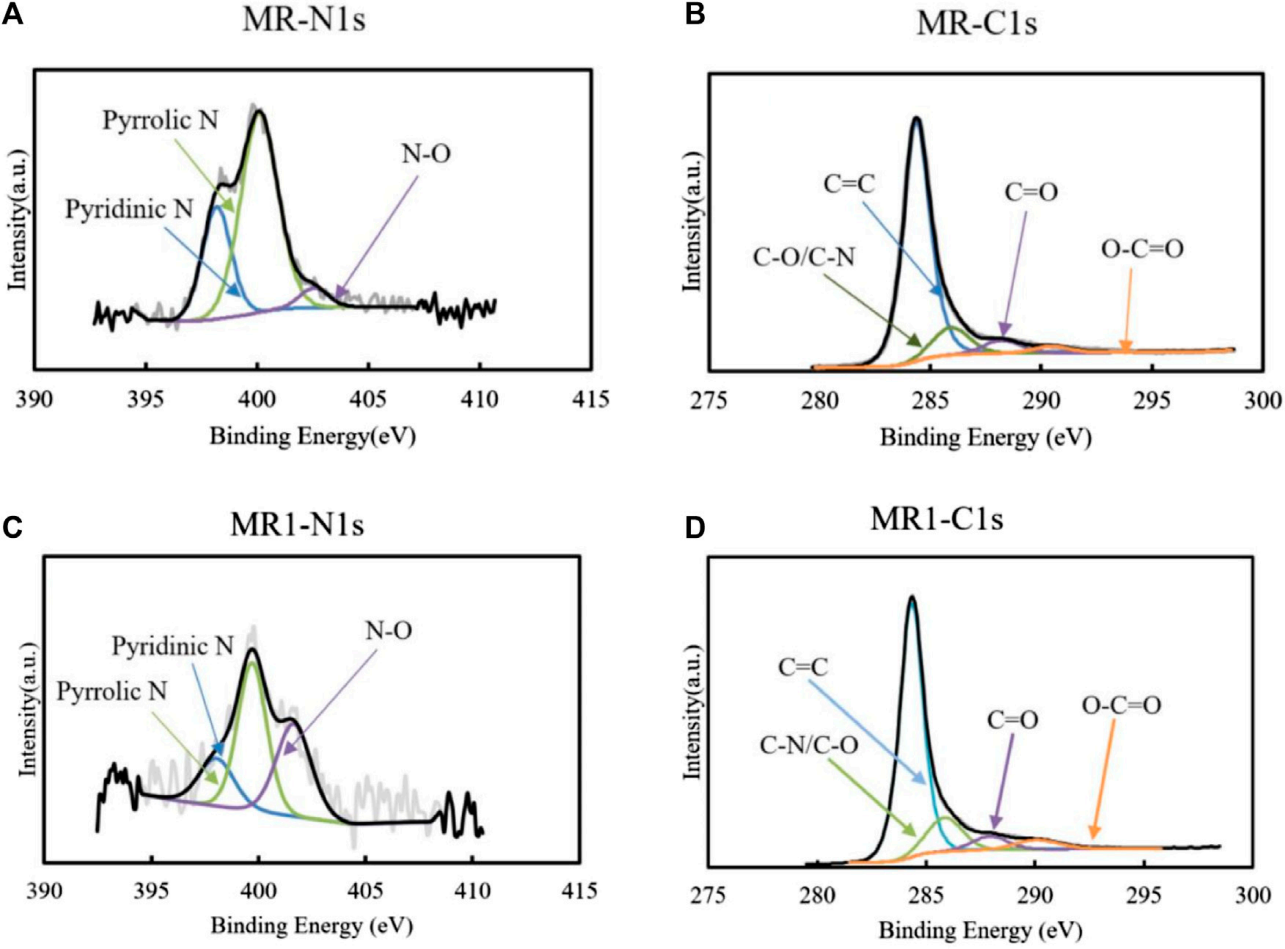
N1s and C1s XPS survey spectra of MR **(A, B)** and MR1 **(C, D)**.

To further evaluate the structure of activated carbon, XPS was used to analyze the surface of MR1. The C1s spectrum of MR1 exhibited the following peaks: C=C (284.3 eV), C=O (287.9 eV), C−N/C−O (285.9 eV), and O−C=O (290.0 eV) (Diyuk et al., 2021) (Figure 2; Table 2). NaOH pretreatment and phosphoric acid activation decreased the C=C and C=O of activated carbon while increasing the C-O/C−N and O-C=O. Similarly, the N1s spectrum was subdivided into four distinct peaks at 398.0, 399.7, and 401.6 eV, each of which corresponds to the presence of pyridinic N, pyrrolic N, or N−O, respectively (Dou et al., 2023). The elevated nitrogen content in the activated carbon resulted in an associated increase in the concentrations of pyridinic nitrogen, pyrrolic nitrogen, and N−O. Pyrrolic N comprised 46.7% of the overall nitrogen content in MR1, whereas prior studies have reported that it contributes to the adsorption of pollutants via activated carbon. Supplementary Figure S3 shows two Raman spectral characteristic peaks of MR1 at 1,349 and 1,586 cm^−1^. These peaks are identified as disordered carbon (D) and graphitic carbon (G), respectively.

**TABLE 2:** Relative surface atomic ratios of MR and MR1.

	C=C	C−O/C−N	C=O	O−C=O	Pyridinic N	Pyrrolic N	N−O
MR	68.8	9.90	4.77	2.29	0.058	0.132	0.010
MR1	56.2	10.7	4.38	5.40	0.468	1.220	0.915

### 3.2 Adsorption experiments and kinetics

**FIGURE 3:**
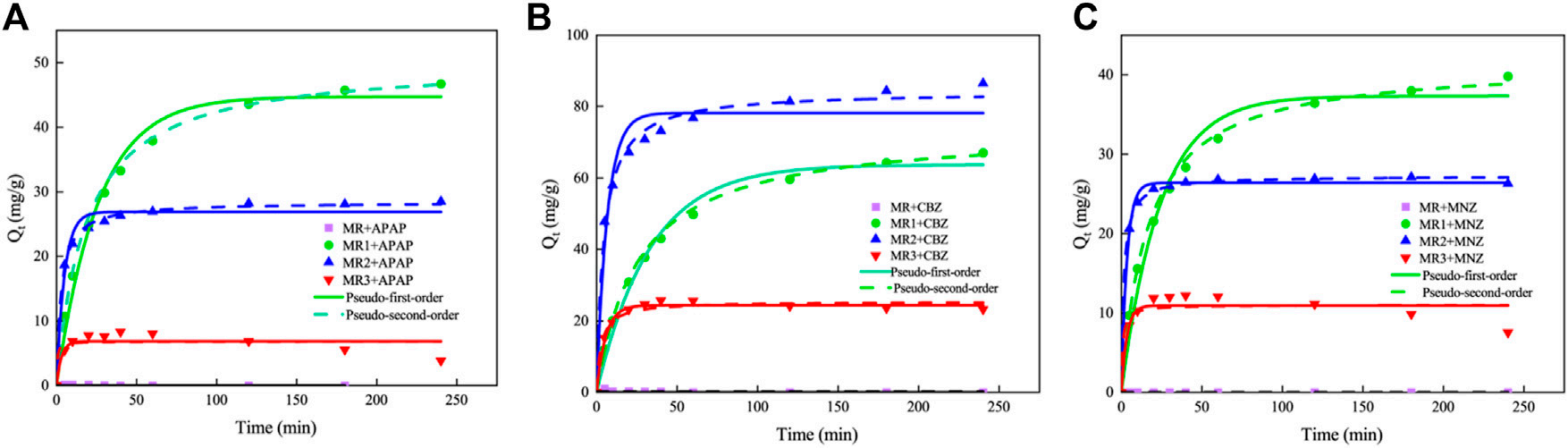
**(A)** APAP, **(B)** CBZ, and **(C)** MZN adsorption kinetics on MR, MR1, MR2, and MR3.

The influence of adsorbent contact time on the adsorption capacity for APAP, CBZ, and MNZ is depicted in Figure 3. Table 3 compares the adsorption of APAP, CBZ, and MNZ on *Lentinus edodes* residue-based activated carbon materials. Without phosphoric acid activation, activated carbon MR has a negligible adsorption capacity for three organic contaminants. However, MR1, MR2, and MR3 all exhibited good adsorption performance for the three organic pollutants. Within 50 min, the adsorption capacity of all activated carbons for the three contaminants increased rapidly and then gradually reached saturation. A pseudo-first- and -second-order kinetic model was used to investigate the probable adsorption kinetic mechanism. The *R* ^2^ value for the pseudo-second-order model was greater than 0.95 (Table 4). This indicates the pseudo-second-order model provides a more accurate description of the adsorption of APAP, CBZ, and MNZ on MR1, MR2, and MR3, in comparison to the pseudo-first-order model (Parimelazhagan et al., 2024). These results suggest chemisorption is the dominant mechanism underlying adsorption, as the pseudo-second-order kinetic model applies to strongly coupled chemisorption processes (Zhang T. et al., 2023). Adsorbents in chemisorption facilitate chemical processes by exchanging electrons with one another (Fatih Dilekoglu and Yapici, 2023).

**TABLE 3:** Kinetic parameters for APAP, CBZ and MNZ adsorption on MR1, MR2 and MR3.

	Pseudo-first-order			Pseudo-second-order		
	Q_e_ (mg g^−1^)	k_1_ (min^−1^)	*R* ^2^	Q_e_ (mg g^−1^)	k_2_ (g mg min^−1^)	*R* ^2^
APAP on MR1	46.86	0.024	0.97	50.76	0.0010	0.99
APAP on MR2	29.14	0.015	0.84	28.82	0.1029	0.99
APAP on MR3	10.89	0.04	0.81	4.21	0.0148	0.95
CBZ on MR1	70.47	0.013	0.99	74.63	0.0005	0.99
CBZ on MR2	89.83	0.183	0.99	85.47	0.0024	0.99
CBZ on MR3	26.55	0.09	0.98	2.35	5.19	0.99
MNZ on MR1	40.78	0.016	0.97	42.55	0.0012	0.99
MNZ on MR2	5.27	0.01	0.98	26.88	0.0399	0.99
MNZ on MR3	13.57	0.57	0.85	8.10	0.0112	0.97

**TABLE 4:** Langmuir and Freundlich adsorption isotherm parameters of APAP, CBZ and MNZ at 298 K in aqueous solutions.

	Langmuir model			Freundlich model		
	X_m_ (mg/g)	K_L_ (L/mg)	*R* ^2^	1/n	K_F_	*R* ^2^
APAP	446.5	144.5	0.94	0.81	4.85	0.95
CBZ	221.2	8.97	0.97	0.56	29.2	0.97
MNZ	137.5	26.6	0.98	0.55	10.3	0.99

### 3.3 Adsorption isotherm

**FIGURE 4:**
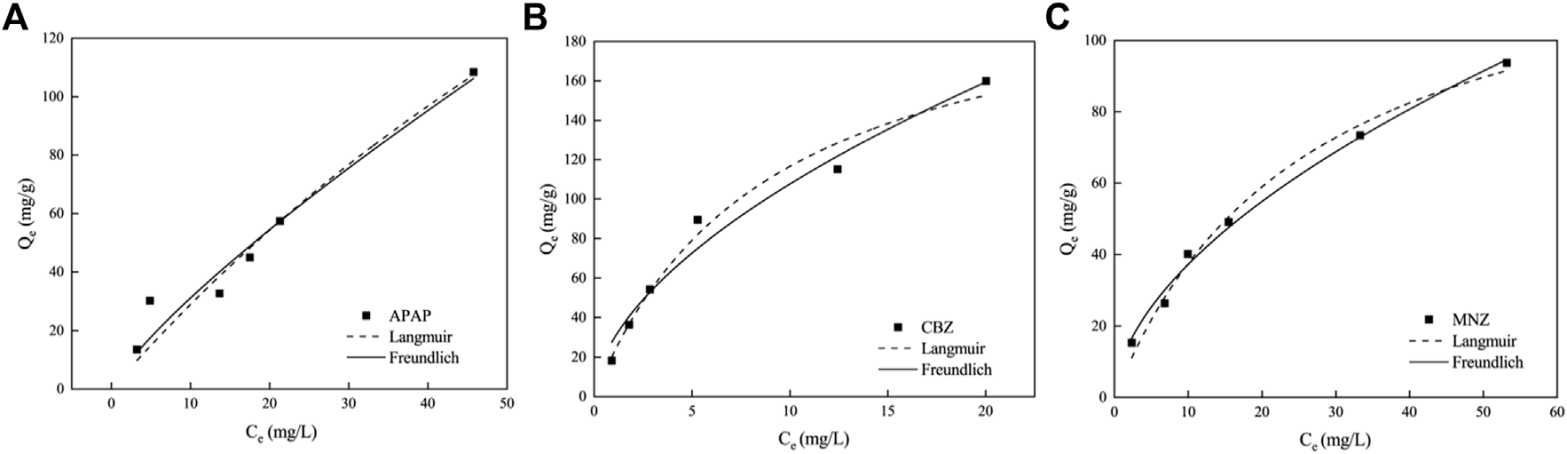
Adsorption isotherms of **(A)** APAP, **(B)** CBZ, and **(C)** MZN on MR1.

To examine the adsorption behavior between MR1 and APAP, CBZ, and MNZ, nonlinear fitting analysis was performed using Langmuir and Freundlich isotherm models on the experimental data. At all three temperatures, the *R* ^2^ of the Freundlich model was greater than 0.99, demonstrating the model adequately describes the MR1 adsorption process on APAP, CBZ, and MNZ (Figure 4; Table 4). The surface energy of the adsorbent is assumed to be irregularly distributed in the Freundlich model, which is an empirical formula (Cardenas et al., 2023). It is typically utilized in low-concentration solutions during the adsorption process. The Freundlich model provided a more accurate fit for the three organic pollutants in this study, owing to their limited solubility in water. The Freundlich model uses the heterogeneity index (n) of the adsorbent to ascertain the adsorption difficulty. Adsorption becomes difficult when 1/n ≥ 1, but it is simple when 0 < 1/n < 1. In this study, 0 < 1/n < 1 indicates MR1 readily adsorbed all three organic pollutants. However, it is also clear that the Langmuir model can represent the adsorption process of MR1 for APAP, CBZ, and MNZ, as the *R* ^2^ value for fitting the adsorption data using the Langmuir model equation was greater than 0.94. This suggests the three organic pollutants adsorb in a near monolayer on the surface of MR1 (Senniappan et al., 2023). As shown in Table 5, the adsorption capacity of MR1 for APAP, CBZ, and MNZ is superior to that of other adsorbents.

**TABLE 5:** Comparison of other similar adsorbents for CBZ, APAP and MNZ removal.

Adsorbent	Adsorbate	Q_max_ (mg/g)	References
Hydrochars and steam activated carbon	CBZ	60	Aghababaei et al. (2023)
Carbon nanotubes	CBZ	130	Lerman et al. (2013)
Oat hulls activated carbon	CBZ	99	Aghababaei et al. (2021)
*Lentinus edodes* residue activated carbon	CBZ	221.17	This work
Spent tea leaves activated carbon	APAP	59.1	Wong et al. (2018)
Corn cob activated carbon	APAP	64.99	Varela et al. (2024)
*Lentinus edodes* residue activated carbon	APAP	446.5	This work
Soybean hulls activated carbon	MNZ	52.42	Schneider et al. (2024)
Modified activated carbon with amine groups	MNZ	66.22	Ahmadfazeli et al. (2021)
*Lentinus edodes* residue activated carbon	MNZ	137.5	This work

### 3.4 Effect of solution pH and activated carbon dosage

**FIGURE 5:**
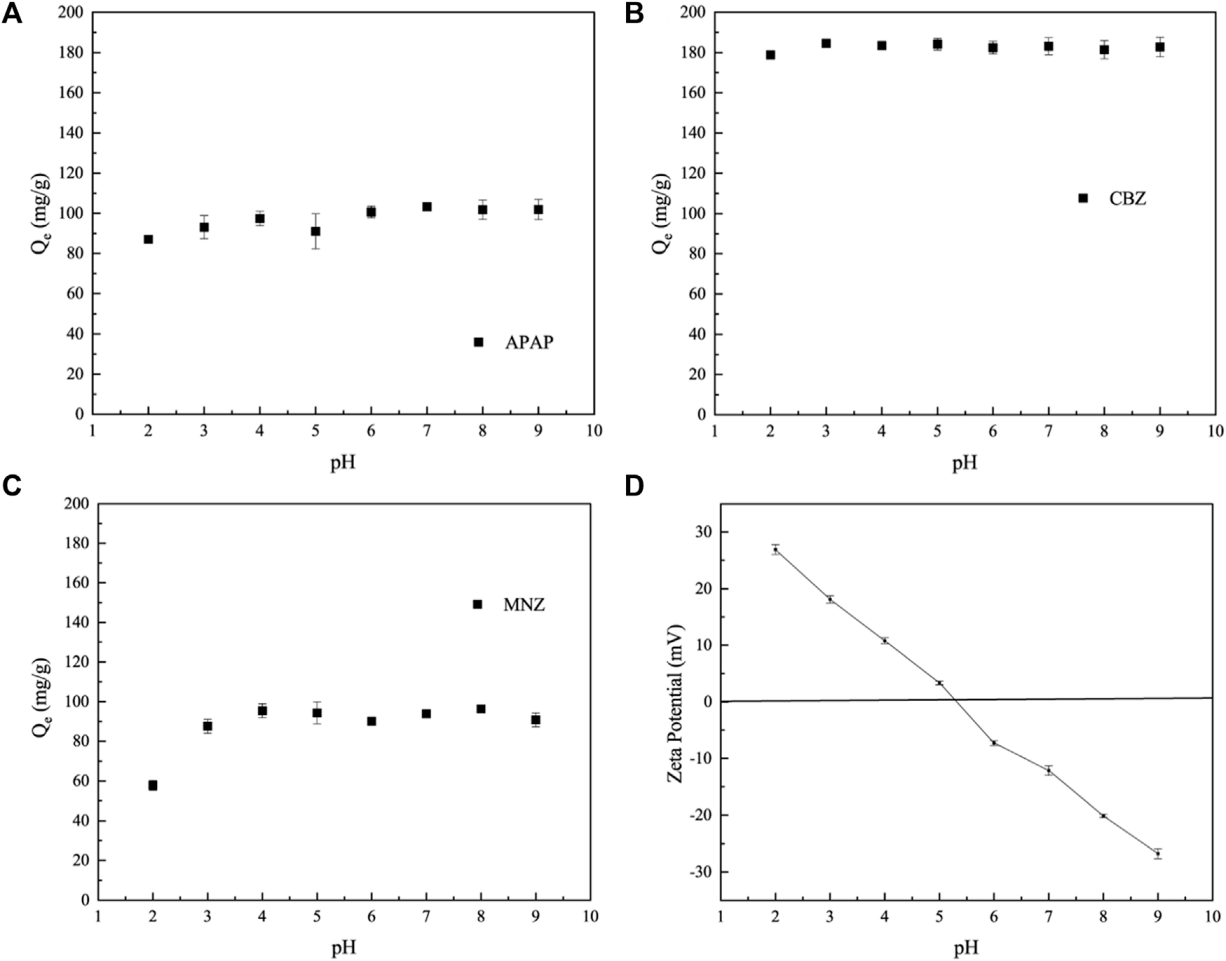
Effect of initial pH on APAP **(A)**, CBZ **(B)** and MNZ **(C)** removal; **(D)** Zeta potential of MR1 at various pH values.

The adsorption performance of MR1 for APAP and CBZ remains basically unchanged, as shown in Figures 5A, B. On the contrary, when the pH level increases from 2 to 3, the ability of MR1 to adsorb MNZ significantly increases, as shown in Figure 5C. In contrast, the adsorptive performance of MR1 towards CBZ and APAP remained largely unchanged. As the pH continued to increase, the adsorption of MNZ, CBZ, and APAP on MR1 initially experienced a modest surge, which was then followed by a slight decline; this change was not significant from a statistical standpoint. Previous studies have indicated that the impact of pH on the adsorption efficiency of active carbon for organic contaminants is linked to the presence of hydroxyl and carboxyl functional groups, which have pKa values ranging from 9.5 to 13 and from 1.7 to 4.7, respectively (Tran et al., 2017; Cheng et al., 2022). The–COOH group is susceptible to deprotonation at pH > 4.7, while the −OH group is susceptible to deprotonation at pH > 9.5. CBZ lacked both carboxyl and hydroxyl functional groups, whereas MNZ and APAP primarily contained hydroxyl functional groups. Hence, within the pH range of 2–9, the adsorption capacity of these three organic pollutants remained largely constant. The present study selected pH = 7 for additional investigation to emulate real-world applications. The surface charge of MR1 measured as zeta-potential is presented in Figure 5D. The pH measurement for MR1 stood at roughly 5.3. This suggests that at pH levels below 5.3, MR1’s surface acquired a positive charge. On the flip side, at pH levels exceeding 5.3, the charge on the surface of the adsorbing agents shifted to a negative polarity.

**FIGURE 6:**
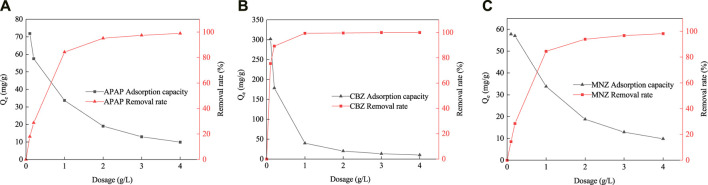
Effect of activated carbon dosage [Adsorption capacity and removal rates of APAP **(A)**, CBZ **(B)** and MNZ **(C)**].

The removal of APAP, CBZ, and MNZ by MR1 increased substantially with increasing dosage before stabilizing (Figure 6). When the dosage of adsorbent was 1 g L^−1^, the removal rates of APAP, CBZ, and MNZ by MR1 were 84.5, 99.3, and 84.2%, respectively. A slight change was observed in the removal rate with increasing dosage of the adsorbent. This phenomenon might arise from an overabundance of MR1 within the solution, which could obstruct the available sites for adsorption, leading to a more incremental enhancement in the adsorption capability (Davoodbeygi et al., 2023).

### 3.5 Regeneration experiment

**FIGURE 7:**
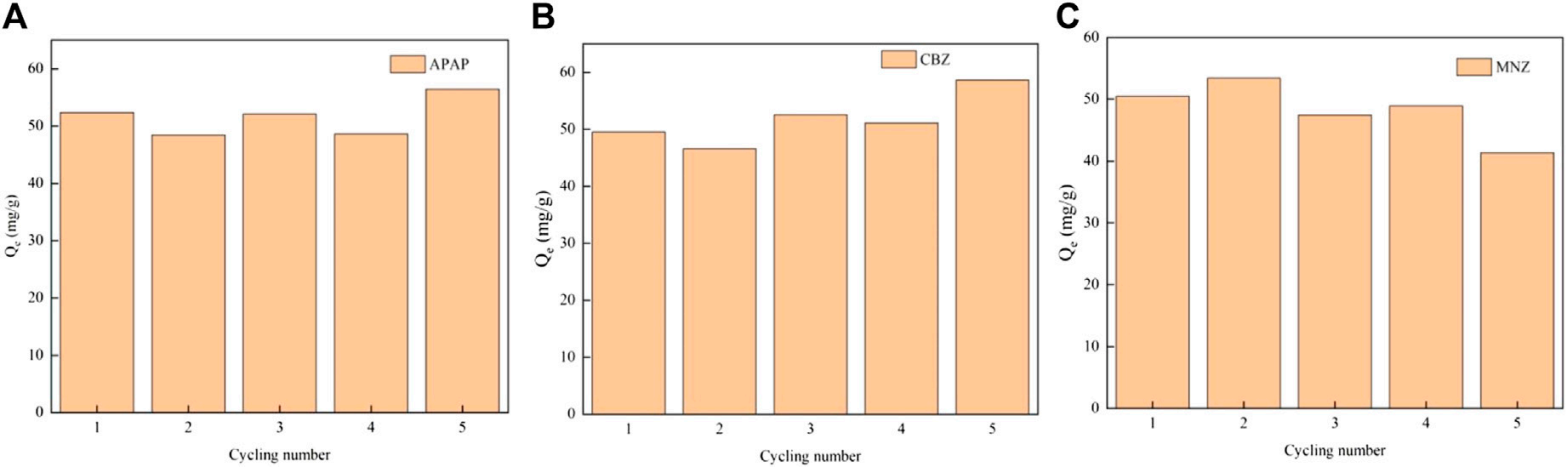
The regeneration adsorptive performance of MR1 after five cycles [Adsorption capacity of APAP **(A)**, CBZ **(B)** and MNZ **(C)**].

The regeneration adsorption performance of MR1 for the three contaminants was very good after five cycles of adsorption and desorption experiments (Figure 7). The adsorption capacity of the three compounds fluctuated as adsorption times increased but remained greater than 95% of the initial adsorption capacity. Methanol has been shown to effectively desorb MR1-activated carbon. Methanol and PPCPs are both organic molecules with similar molecular structures, and PPCPs are highly soluble in methanol. When they collide with distinct phase interfaces in an ultrasonic cleaner, enormous compressive force is produced. Tiny cavitation bubbles appear as the wave rebounds. When the cavitation bubbles burst, the temperature and pressure at the explosion site increase dramatically, allowing energy to be transferred to the adsorbed material. This accelerates the thermal mobility and isolates PPCPs from the activated carbon surface. At the same time, microbubbles can form additional microporous structures while maintaining the outstanding adsorption capacity of activated carbon. Overall, *Lentinus edodes* residue carbon exhibited a reasonably high and stable removal efficiency of PPCPs, as well as regeneration capability. Meanwhile, we observed minimal differences in the adsorption capacity of MR1 for APAP, CBZ, and MNZ between distilled water and tap water, with a slight reduction in the tap water setting, as depicted in Supplementary Figure S5. This suggests that MR1 holds significant practical application value. Supplementary Figure S6 demonstrates that MR1 maintains a robust porous structure after regeneration, demonstrating its stable structure and capability for repeated regeneration.

### 3.6 Possible adsorption mechanism

**FIGURE 8:**
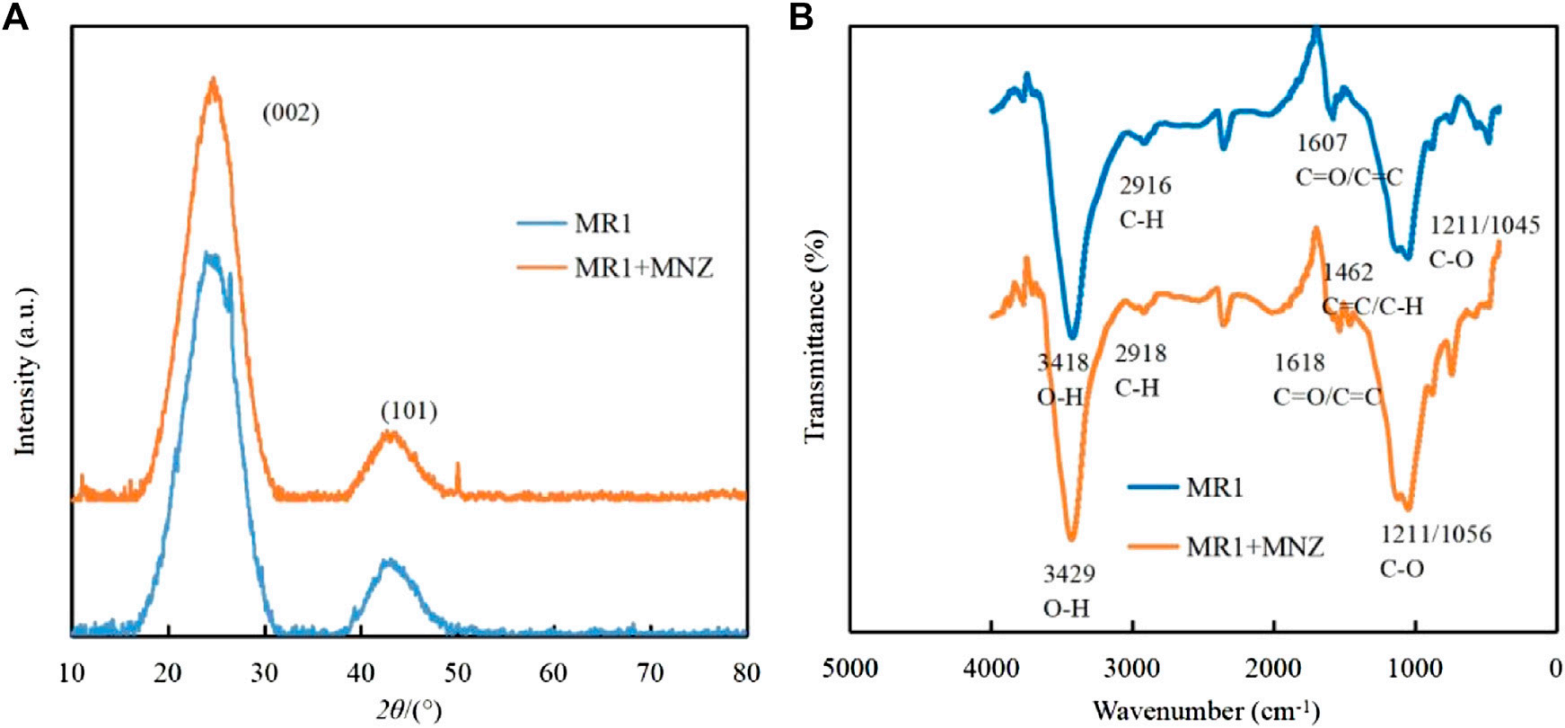
**(A)** XRD spectra of MR1 and MR1 with adsorbed MNZ. **(B)** FTIR spectra of MR1 and MR1.

Investigations into the alterations in MR1’s microstructure pre and post adsorption were conducted using FTIR and XRD techniques. As depicted in Figure 8A, the XRD patterns before and after MNZ adsorption on MR1 were examined. Notably, the pronounced peak at 26.5 and a less intense peak at 44.6 were associated with the (002) and (101) crystalline planes of graphite, as noted in the literature (Tian et al., 2020; Venkatesan et al., 2023). The presence of an aromatic graphite structure in the N-doped activated carbon of MR1 enhances its role as a π-electron acceptor, which facilitates the π-π electron donor acceptor (π-π EDA) interactions with organic contaminants that act as π-electron donors (Miao et al., 2022). By applying the Bragg equation, the spacing between layers of MR1 before and after MNZ adsorption was determined, indicating that the adsorption of MNZ on MR1 predominantly takes place via hydrogen bonds or charge transfers on MR1’s surface (Li et al., 2023).

For the activated carbon sample treated with phosphoric acid, the transmission peak at 3,418 cm^−1^ is attributed to the O−H stretching vibrational mode (Figure 8B). The peak at 2,916 cm^−1^ is ascribed to the C−H stretching vibrations typically found in aliphatic or cycloalkane structures (Kodali et al., 2021). The relatively low intensity of this peak suggests a demethylation reaction during the phosphoric acid activation process. The transmission peak at 1,618 cm^−1^, which overlap, represent a complex mixture predominantly comprising ester functionalities, such as carbonyl C=O and aromatic C=C structures. The peaks at 1,121 cm^−1^ and 1,045 cm^−1^ are principally associated with the C−O stretching vibrational mode. Upon the adsorption of MNZ onto the activated carbon, notable spectral changes are observed, including a blue shift of the O−H transmission peak from 3,418 cm^−1^–3,429 cm^−1^, accompanied by an alteration in peak morphology. This suggests the inclusion of MNZ modifies the hydrogen bonding interactions of the hydroxyl groups within the sample. Additionally, the transmission peaks corresponding to the C=O at 1,607 cm^−1^ and the C−O groups at 1,045 cm^−1^ exhibit significant shifts, suggesting oxygen-containing functional groups, such as carboxyl groups, play a pivotal role as binding sites for MNZ. The emergence of a new mixed transmission peak at 1,462 cm^−1^, indicative of C=C planar vibrations and C−H stretching vibrations, could be attributed to the structural integration of MNZ. These spectral characteristics substantiate the occurrence of both physical and chemical adsorption mechanisms in the removal of MNZ by activated carbon. Oxygen-containing functional groups, particularly hydroxyl and carboxyl groups, appear to serve as crucial crosslinking sites in this process.

**FIGURE 9:**
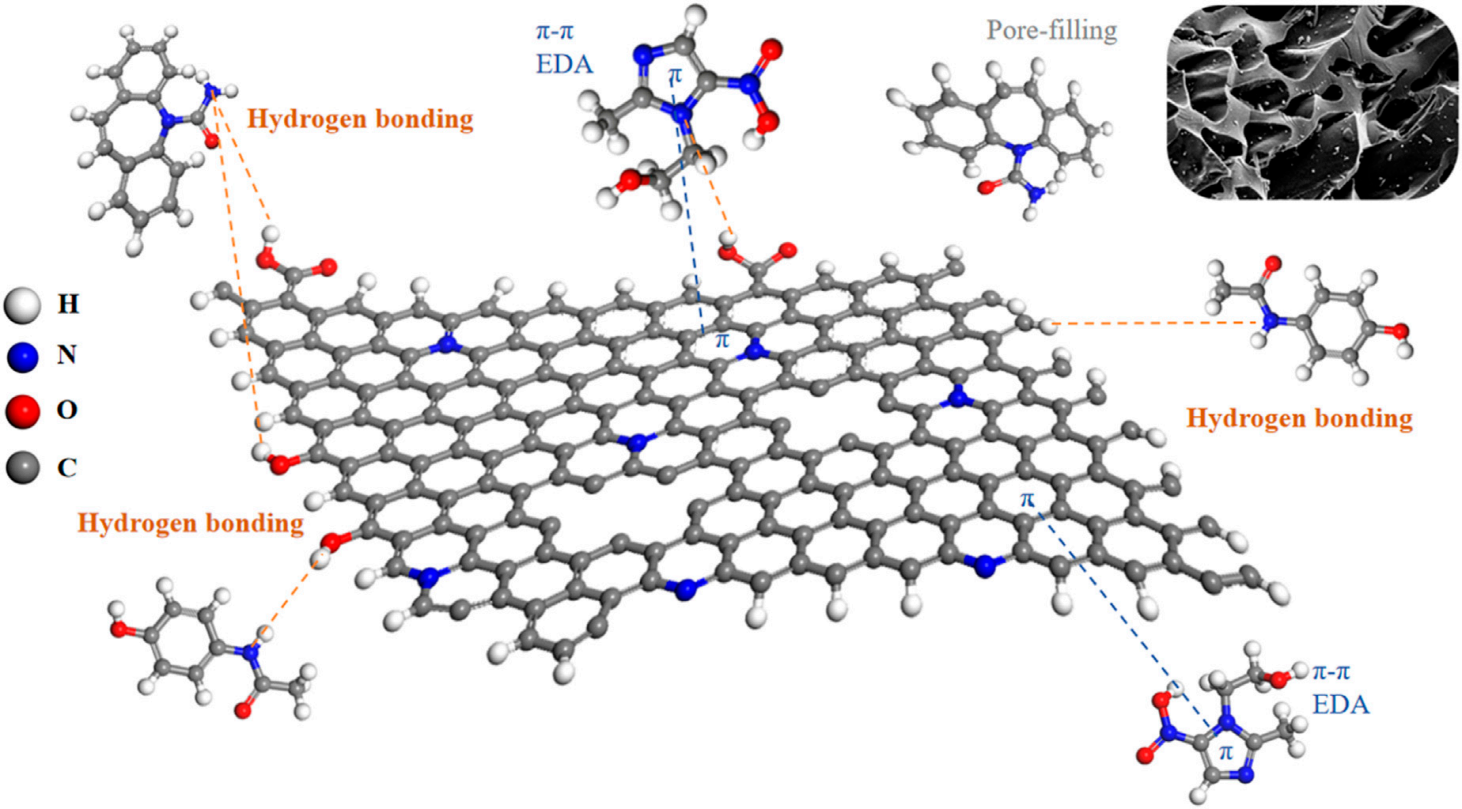
The proposed adsorption mechanisms of APAP, CBZ and MNZ onto MR1.

The following adsorption mechanism was hypothesized based on the investigation of adsorption kinetics, adsorption isotherms, XRD, and FTIR: (1) The surface of MR1 has a large specific surface area and a relatively developed pore structure, allowing for surface adsorption and internal pore filling diffusion adsorption of APAP, CBZ, and MNZ; (2) C=O on the MR1 surface can form hydrogen bonds with hydroxyl or amino groups on APAP, CBZ and MNZ, while OH present on MR1 can also form hydrogen bonds with C=O on APAP and MNZ molecules; (3) The Lewis acid-base effect can occur when pyrrole N on the surface of MR1 binds to Lewis acid sites (−OH) on APAP, CBZ, and MNZ as Lewis base sites (−NH_2_, −NH−). The possible adsorption mechanism was shown in Figure 9.

## 4 Conclusion

This study successfully utilized phosphoric acid activation to synthesize nitrogen-doped mesoporous activated carbon (MR1) from *Lentinus edodes* residue. The incorporation of nitrogen and the development of a mesoporous structure endowed MR1 with the capability to adsorb acetaminophen (APAP), carbamazepine (CBZ), and metronidazole (MNZ). The superior adsorption performance of MR1 can be ascribed to the synergistic effects of hydrogen bonding, π-π interactions, and Lewis acid-base interactions, which facilitate the capture of these organic pollutants. Moreover, MR1 demonstrated stability and effectiveness across a broad pH range (2–10) and under conditions of high ionic strength, maintaining a high recovery efficiency. These attributes underscore the versatility of MR1 and highlight its potential as an environmentally sustainable adsorbent that can be used for water purification. Our findings suggest that MR1 is a promising candidate for addressing the challenges of organic pollutant removal in various water treatment scenarios.

## Data Availability

The raw data supporting the conclusion of this article will be made available by the authors, without undue reservation.
