# Supplementary material for: Nitrogen-doped mesoporous activated carbon from Lentinus edodes residue: an optimized adsorbent for pharmaceuticals in aqueous solutions
Source: Front Chem. 2024 Jun 20;12:1419287. doi: 10.3389/fchem.2024.1419287 (PMC11222600; doi:10.3389/fchem.2024.1419287)
Supplement: Supplementary file 1 [file DataSheet1.docx]

Supplementary Material


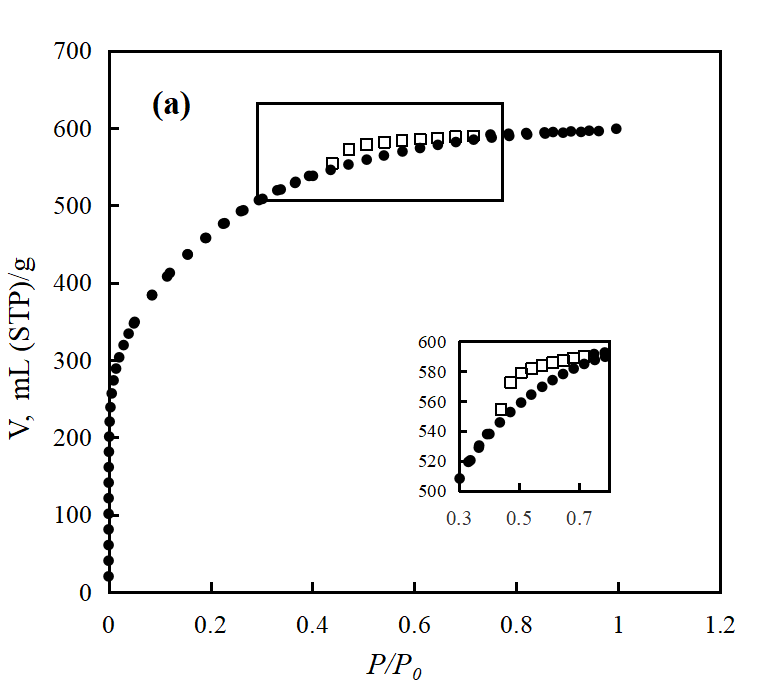


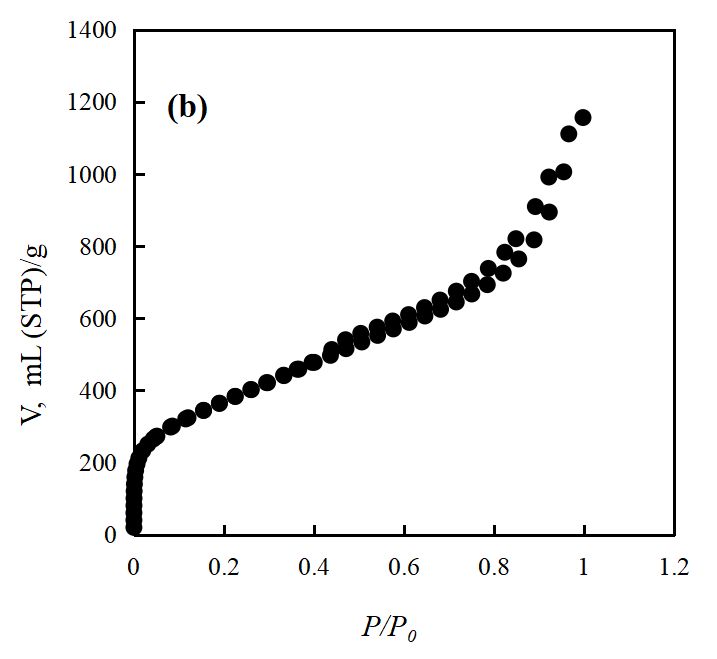

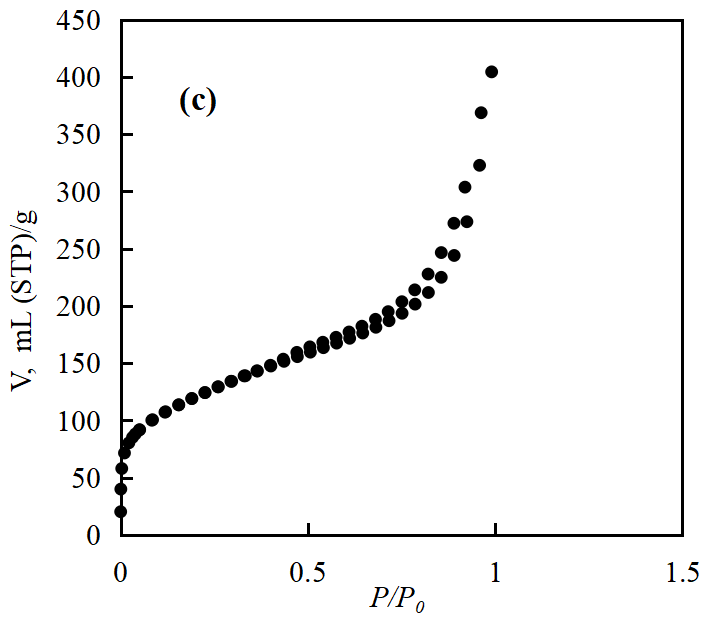


Figure S1. Nitrogen adsorption isotherm of MR1 (a), MR2 (b), and MR3(c).

O1s

N1s

C1s

O1s

N1s

C1s

Figure S2. XPS spectra of MR(a) and MR1(b).

Figure S3. Raman spectra of MR1.


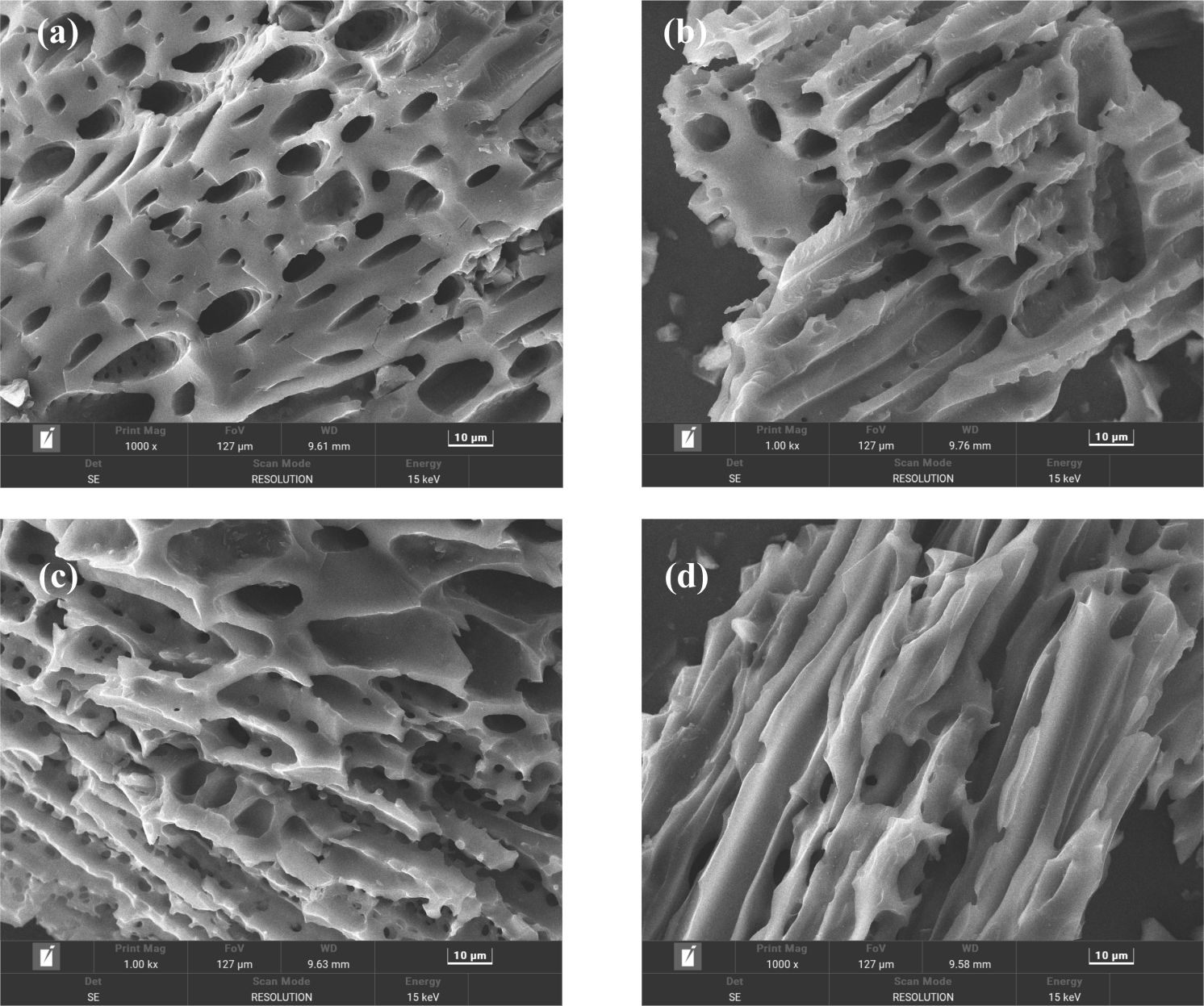


Figure S4. SEM images of MR1 before adsorption (a), and after adsorption of APAP (b), CBZ (c), and MZN (d).

Figure S5. MR1's adsorption capacity on APAP, CBZ, and MNZ in both distilled and tap water. ()


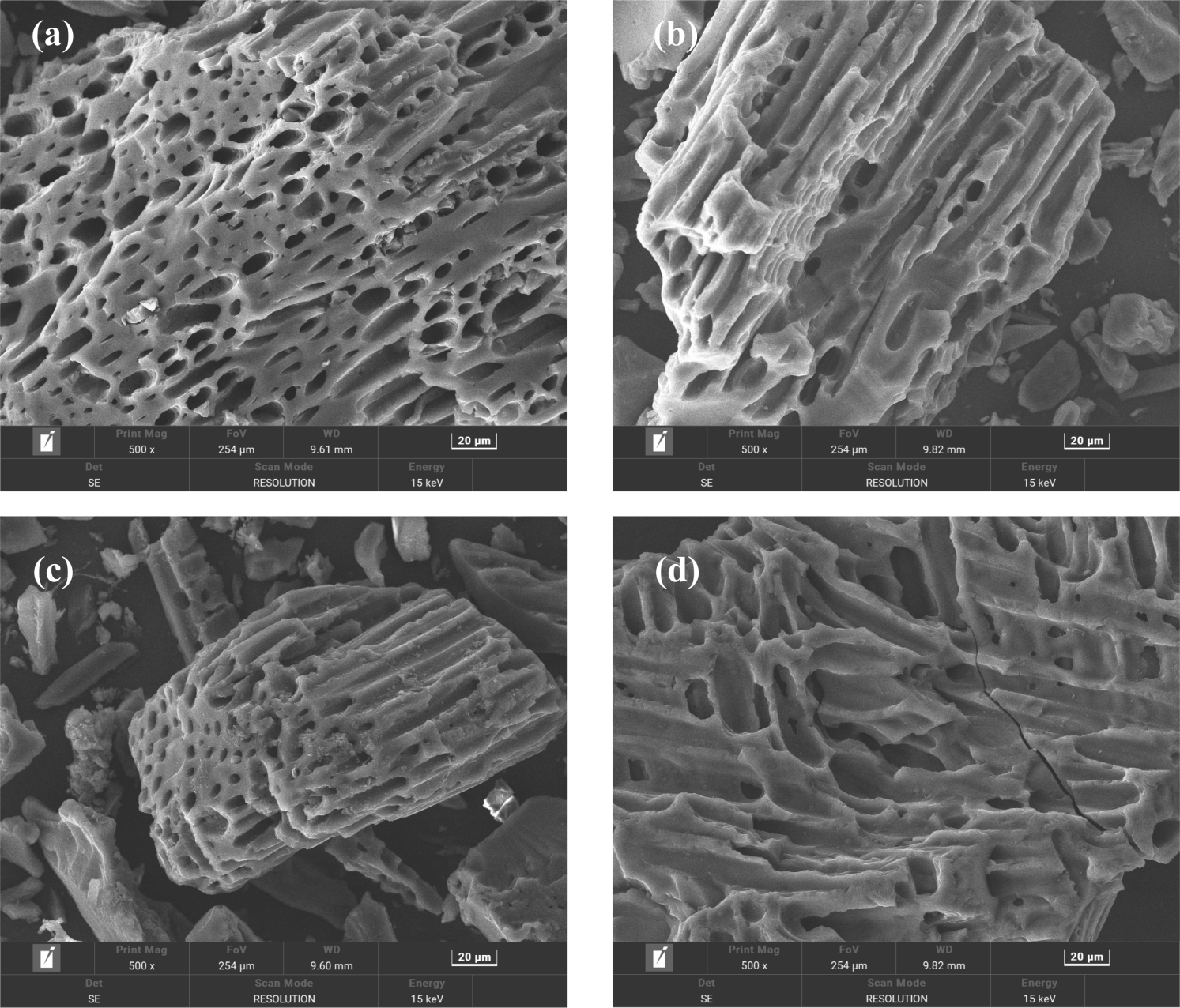


Figure S6. SEM images of MR1 before adsorption (a), and after regeneration of APAP (b), CBZ (c), and MZN (d).


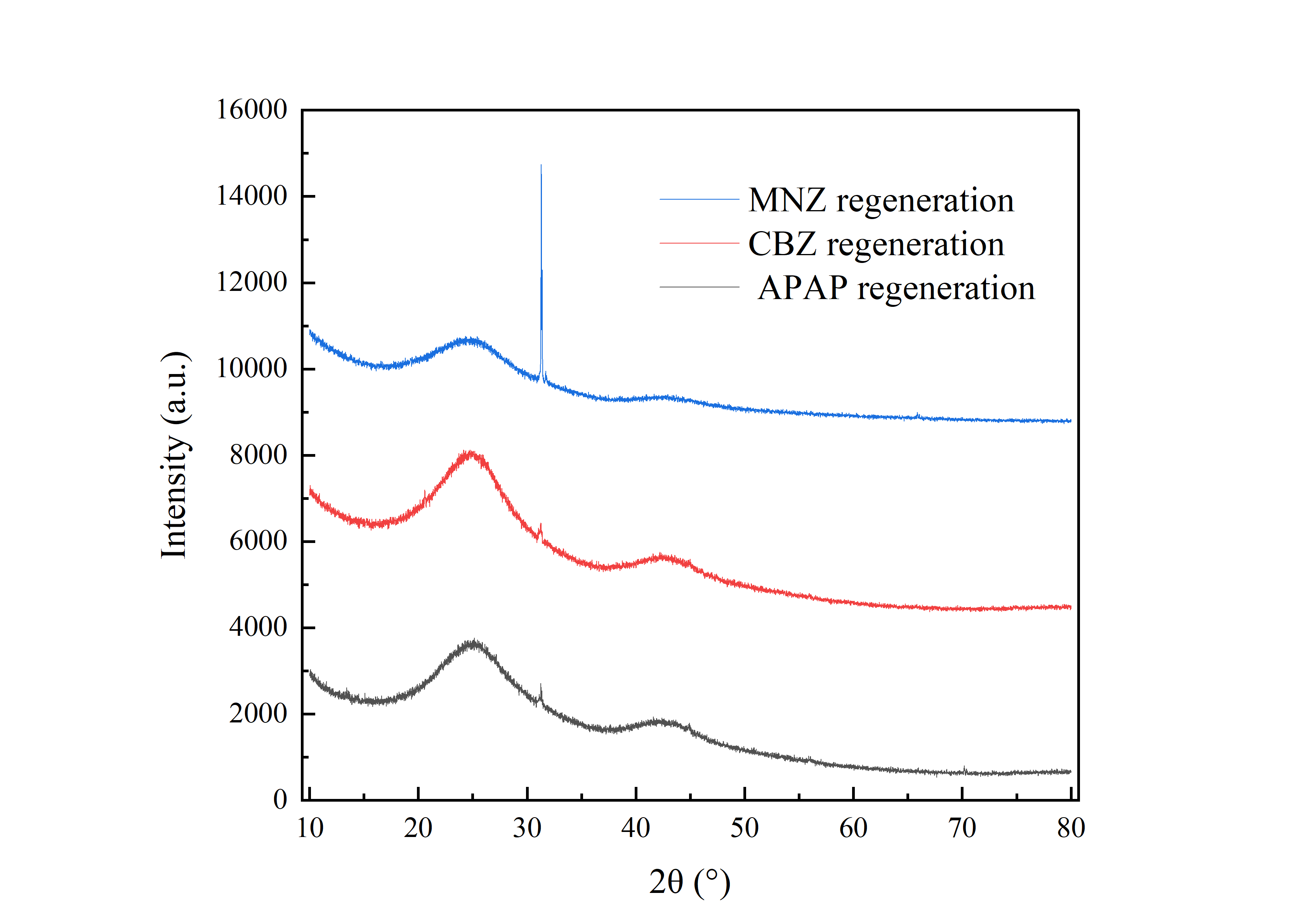


Figure S7. XRD spectra of of MR1 after regeneration of APAP, CBZ, and MZN.
